# Using single-worm RNA sequencing to study *C. elegans* responses to pathogen infection

**DOI:** 10.1186/s12864-022-08878-x

**Published:** 2022-09-14

**Authors:** Archer J. Wang, Phillip Wibisono, Blake M. Geppert, Yiyong Liu

**Affiliations:** 1 Genomics Core, Washington State University, Spokane, WA USA; 2 Department of Translational Medicine & Physiology, Elson S. Floyd College of Medicine, Washington State University, Spokane, WA USA

**Keywords:** Single-worm RNA sequencing, Low-input RNA sequencing, Library preparation, Library quality metrics, Pathogen infection, *Caenorhabditis elegans*, Immune response

## Abstract

**Background:**

*Caenorhabditis elegans* is an excellent research model whose populations have been used in many studies to address various biological questions. Although worm-to-worm phenotypic variations in isogenic populations have been persistently observed, they are not well understood and are often ignored or averaged out in studies, masking the impacts of such variations on data collection and interpretation. Single-worm RNA sequencing that profiles the transcriptomes of individual animals has the power to examine differences between individuals in a worm population, but this approach has been understudied. The integrity of the starting RNA, the quality of the library and sequence data, as well as the transcriptome-profiling effectiveness of single-worm RNA-seq remain unclear. Therefore, more studies are needed to improve this technique and its application in research.

**Results:**

In this study, we aimed to develop a single-worm RNA-seq method that includes five steps: worm lysis and RNA extraction, cDNA synthesis, library preparation, sequencing, and sequence data analysis. We found that the mechanical lysis of worms using a Qiagen TissueLyser maintained RNA integrity and determined that the quality of our single-worm libraries was comparable to that of standard RNA-seq libraries based on assessments of a variety of parameters. Furthermore, analysis of pathogen infection-induced gene expression using single-worm RNA-seq identified a core set of genes and biological processes relating to the immune response and metabolism affected by infection. These results demonstrate the effectiveness of our single-worm RNA-seq method in transcriptome profiling and its usefulness in addressing biological questions.

**Conclusions:**

We have developed a single-worm RNA-seq method to effectively profile gene expression in individual *C. elegans* and have applied this method to study *C. elegans* responses to pathogen infection. Key aspects of our single-worm RNA-seq libraries were comparable to those of standard RNA-seq libraries. The single-worm method captured the core set of, but not all, infection-affected genes and biological processes revealed by the standard method, indicating that there was gene regulation that is not shared by all individuals in a population. Our study suggests that combining single-worm and standard RNA-seq approaches will allow for detecting and distinguishing shared and individual-specific gene activities in isogenic populations.

**Supplementary Information:**

The online version contains supplementary material available at 10.1186/s12864-022-08878-x.

## Background

*Caenorhabditis elegans* is a 1-mm-long roundworm that has been used as a model organism in biological research for more than half a century. In many studies, experiments were performed on isogenic worm populations. Although worm-to-worm phenotypic variations in these populations were persistently observed, such variations were either ignored or averaged out in the final measurements. However, some variations are significant and should be taken into account during data collection and interpretation. For example, in aging research, individual wild-type worms may die at as early as 10 days, while some individuals may live up to 30 days, resulting in a 300% difference in lifespan in the same wild-type population [1]. Thus, treatments that extend lifespan should be examined with consideration of this variability. While the exact causes of differences in individuals with the same genetic background are unclear, Perez et al. reported that maternal age generates phenotypic variations within progenies partly due to age-dependent changes in the maternal provisioning of the yolk proteins vitellogenins to embryos [2]. Interestingly, the expression of a transcriptional reporter of heat shock protein gene *hsp-16.2* was used to accurately predict variance in longevity and resistance to stress in isogenic populations [3, 4]. Because the phenotypes of an organism are the output of its physiological state, which in turn is influenced by a variety of factors, including genetic, environmental, and metabolic processes, the causes underlying phenotypic variations between isogenic individuals could be highly plastic and complex. Nonetheless, it is generally agreed that stochastic factors contribute to the variability between individuals in an isogenic population as well as between cells of the same type within an individual [5]. However, the nature of such stochastic factors remains a mystery. Approaches focusing on single worms or single cells, such as single-worm RNA sequencing and single-cell RNA sequencing, could provide insights into the underlying molecular basis for such variabilities.

In recent years, single-cell RNA-seq has been rapidly developed and widely used to detect cell-to-cell variations in gene activities within a cell population. In *C. elegans*, for example, single-cell RNA-seq has been used to define consensus expression profiles of various cell types at different developmental stages [6, 7]; examine the connectivity and function of individual neurons, including rare neuron types [8, 9]; and map the effects of expression quantitative trait loci at cellular resolution [10]. These advances have enabled researchers to investigate the internal interactions and differences within a cell population, deconvolving the cellular heterogeneity in tissues. In *C. elegans*, single-worm RNA-seq that profiles the transcriptome of the whole animal may have the power to probe individual-to-individual variations within a worm population. Indeed, Dillman and colleagues have adapted a single-cell RNA-seq protocol, Smart-seq2 [11], for single-worm RNA-seq and have analyzed the pathogenesis of individual insect-parasitic nematodes, *Steinernema carpocapsae*, as well as individual embryos and L1 larvae from two *Steinernema* and two *Caenorhabditis* species including *C. elegans* [12–15]. Despite this progress, the integrity of the starting RNA, the quality of the library and sequence data, and the transcriptome-profiling effectiveness of single-worm RNA-seq still remain unclear. Overall, this emerging technique holds the promise of examining differences between individuals in an isogenic population, but more studies are needed to characterize and improve its usefulness for wide application in research.

Compared to standard RNA-seq that uses RNA from hundreds to thousands of worms as input, single-worm RNA-seq faces two major technical challenges: low input RNA from a single worm and low accessibility of that RNA due to the nematode’s thick surrounding cuticle. While the limited amount of input RNA can be overcome by adapting library preparation methods from single-cell RNA-seq, the harsh chemical lysis condition commonly used to penetrate the worm cuticle and access the RNA could potentially cause RNA degradation [12–15]. In the current study, we tested three different conditions for the mechanical lysis of worms and found an optimal condition to maintain RNA integrity. Using RNA isolated from single worms as input, we synthesized cDNA, constructed sequencing libraries, and then evaluated the quality of our libraries by comparing them to standard RNA-seq libraries that were made with 2.5 µg of total RNA using the TruSeq Stranded mRNA Library Preparation Kit (Illumina) [16]. Overall, the key parameters of our single-worm libraries, including alignment rate, rRNA content, read coverage over the position of transcripts, and duplication rate, were comparable to or slightly below those of standard libraries. The concordance of gene expression quantification was less consistent between replicates in single-worm RNA-seq than in standard RNA-seq, possibly because the biological replicates in the former were different individual worms with the same genetic background while the biological replicates of the later were pooled samples that masked individual differences. When applied to the analysis of differentially expressed genes (DEGs) induced by pathogen infection, the single-worm RNA-seq method identified the core set of DEGs and biological processes affected by infection. These results demonstrate the effectiveness of our single-worm RNA-seq method in transcriptome profiling and the usefulness of its application to address biological questions.

## Results

### Overview of the single-worm RNA-seq workflow

**Fig. 1:**
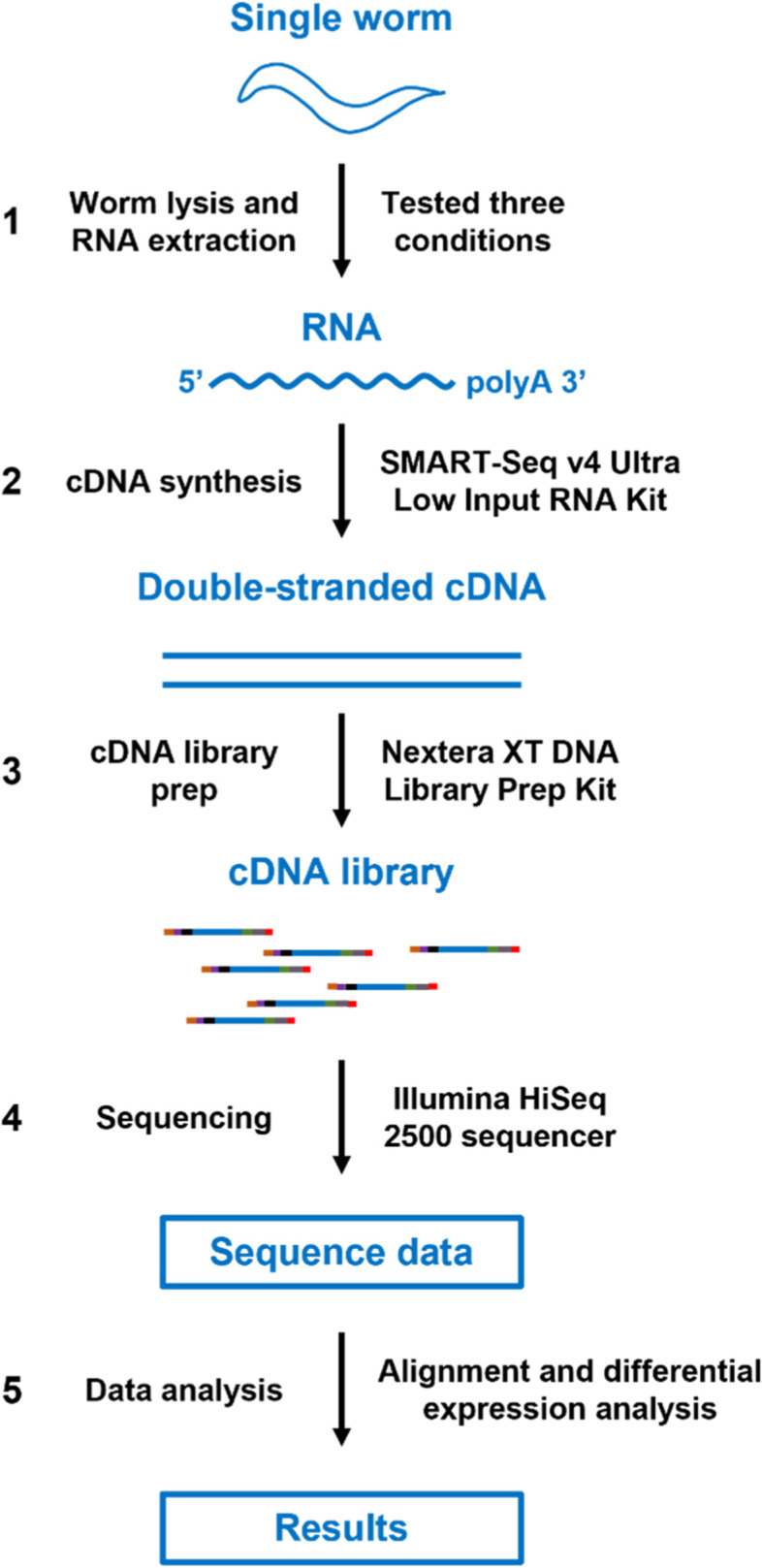
Scheme of the single-worm RNA-seq workflow. The workflow includes five steps: worm lysis and RNA extraction, cDNA synthesis using the SMART-Seq v4 Ultra Low Input RNA Kit, cDNA library prep using the Nextera XT DNA Library Prep Kit, sequencing, and sequence data analysis including alignment and differential gene expression analysis

The workflow of our single-worm RNA-seq method includes five major steps, namely, worm lysis and RNA extraction, cDNA synthesis, library preparation, sequencing, and sequence data analysis. The scheme of our workflow is depicted in Fig. 1. Briefly, we tested three different conditions for the mechanical lysis of worms prior to RNA extraction and chose the condition that generated the highest quality RNA for use in downstream procedures. For cDNA synthesis, the SMART-Seq v4 Ultra Low Input RNA Kit (Takara Bio) was used because of its high sensitivity and its ability to generate full-length cDNA. The cDNA was then made into sequencing-ready libraries using the Nextera XT DNA Library Prep Kit (Illumina). Sequencing was performed on an Illumina HiSeq 2500 sequencer to produce a sufficient number of paired-end reads. The resulting sequence data were then subjected to alignment and differential gene expression analyses. At each step, the results of the single-worm RNA-seq method were compared to those of the standard RNA-seq method by analyzing key parameters. Five biological replicates of four group samples were used for RNA-seq analysis (Table S1). The four group samples were single and bulk worms with or without *Pseudomonas aeruginosa* infection. The RNA-seq analysis of the single-worm samples was conducted in the current study. The RNA-seq analysis of the bulk samples was conducted by us in a previous study [16] and used here as a standard to evaluate the single-worm RNA-seq analysis. Below we describe the five major steps in detail.

### Worm lysis and RNA extraction

**Fig. 2:**
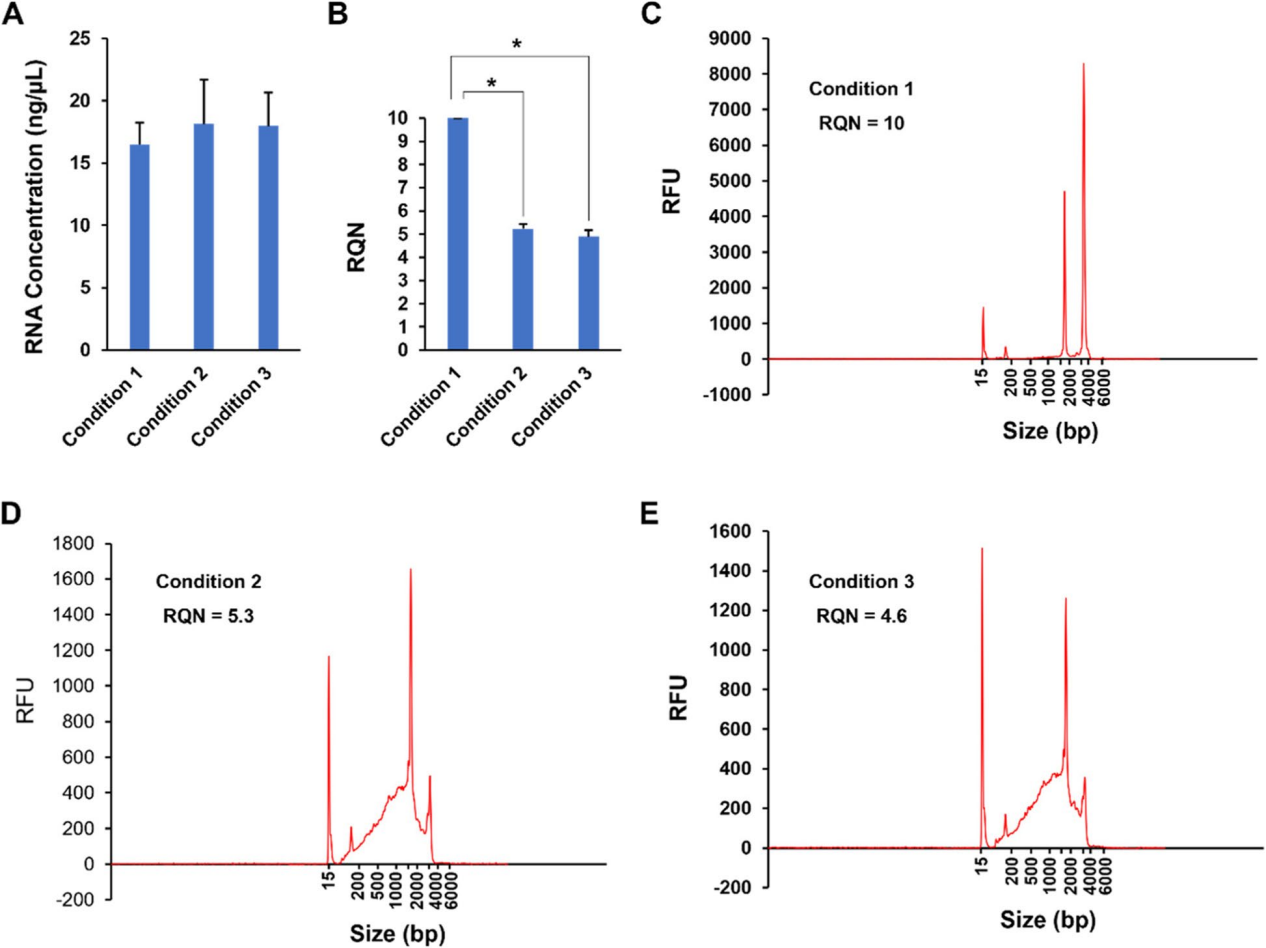
Testing three conditions for the mechanical lysis of worms. Thirty worms per group were mechanically lysed under three different conditions (Table 1), followed by RNA extraction using an RNeasy Mini Kit. **A** RNA concentrations were measured using a Qubit fluorometer. Each bar in the histogram represents the mean value of three replicates. Error bars represent standard deviation. **B** RNA integrity was assessed using a Fragment Analyzer and is expressed as an RNA Quality Number (RQN). An RQN of 10 equates to a perfect RNA sample without any degradation products, and an RQN of 1 indicates a completely degraded sample. Numbers in-between are used to indicate progressing degradation states of the RNA sample. Each bar in the histogram represents the mean value of three replicates. Error bars represent standard deviation. An asterisk (*) denotes a significant difference (*p* < 0.05) between two conditions. **C** Representative profile of RNA extracted under Condition 1. RFU stands for Relative Fluorescence Units. **D** Representative profile of RNA extracted under Condition 2. **E** Representative profile of RNA extracted under Condition 3

In the single-worm RNA-seq method developed by Dillman and colleagues [14, 15], a worm was cut, chemically lysed in a lysis buffer, and then subjected to reverse transcription using SMART-Seq2 reagents. Because the SMART reagents work best with isolated RNA as input [11] and the harsh lysis buffer used to chemically penetrate the worm’s thick cuticle could potentially degrade RNA molecules, we wanted to mechanically break worms, isolate RNA, and then use that RNA as input in our single-worm RNA-seq protocol. To this end, we tested three different conditions for the mechanical lysis of worms prior to RNA extraction using the RNeasy Mini Kit (Qiagen). These conditions were (1) shearing worms with a TissueLyser (Qiagen) without prior worm processing, (2) incubating worms in a lysis buffer followed by shearing with a TissueLyser, and (3) incubating worms in a lysis buffer followed by shearing with a QIAshredder spin column (Qiagen) (Table 1). Because it is difficult to check RNA quality and quantity from a single worm due to the limited amount of RNA, we carried out our testing with 30 worms per group under each condition. Our results showed that while all three conditions produced similar amounts of RNA (Fig. 2A), shearing with a TissueLyser without prior processing (Condition 1 in Table 1) generated the highest quality RNA with an RNA Quality Number (RQN) of 10 (Fig. 2, B and C). An RQN of 10 is the highest quality score possible and indicates no RNA degradation, suggesting that RNA integrity was well preserved under Condition 1. This integrity was comparable to or higher than that of RNA isolated using the standard RNA-seq protocol, which had RQNs of 8 ~ 10 [16]. In contrast, samples generated using Conditions 2 and 3 (incubation in a lysis buffer prior to shearing) had degraded RNA with RQNs around 5 (Fig, 2 B, D and E). These results indicate that incubating in a lysis buffer similar to the one used by Dillman and colleagues [14, 15] causes RNA degradation, and that mechanical lysis without the use of a harsh lysis buffer can produce RNA of high quality. Therefore, we chose Condition 1 for isolating total RNA from single worms and then used this RNA for our downstream RNA-seq protocols.

**Table 1:** Three conditions tested for the mechanical lysis of worms

Condition	Processing before shearing	Device of shearing
1	No processing	TissueLyser LT^b^
2	Incubated in Lysis Buffer^a^ at 65 °C for 10 min, 85 °C for 1 min, then held at 4 °C in a thermocycler	TissueLyser LT^b^
3	Incubated in Lysis Buffer^a^ at 65 °C for 10 min, 85 °C for 1 min, then held at 4 °C in a thermocycler	QIAshredder spin column^c^

^a ^Lysis Buffer was composed of 50 mM KCl, 10 mM Tris–HCl, pH 8.3, 2.5 mM MgCl_2_, 0.45% Triton X-100, 0.45% Tween-20, 0.11% gelatin (w/v), 100 µg/mL proteinase K, and 200 units of RNasin Ribonuclease Inhibitor

^b ^A TissueLyser LT was used following the “Purification of RNA or Multiple Analytes form Animal and Human Tissues” protocol in the manufacturer’s manual

^c ^The QIAshredder spin column was centrifuged at 16,873 × *g* for 2 min

### cDNA synthesis, library preparation, and sequencing

**Fig. 3:**
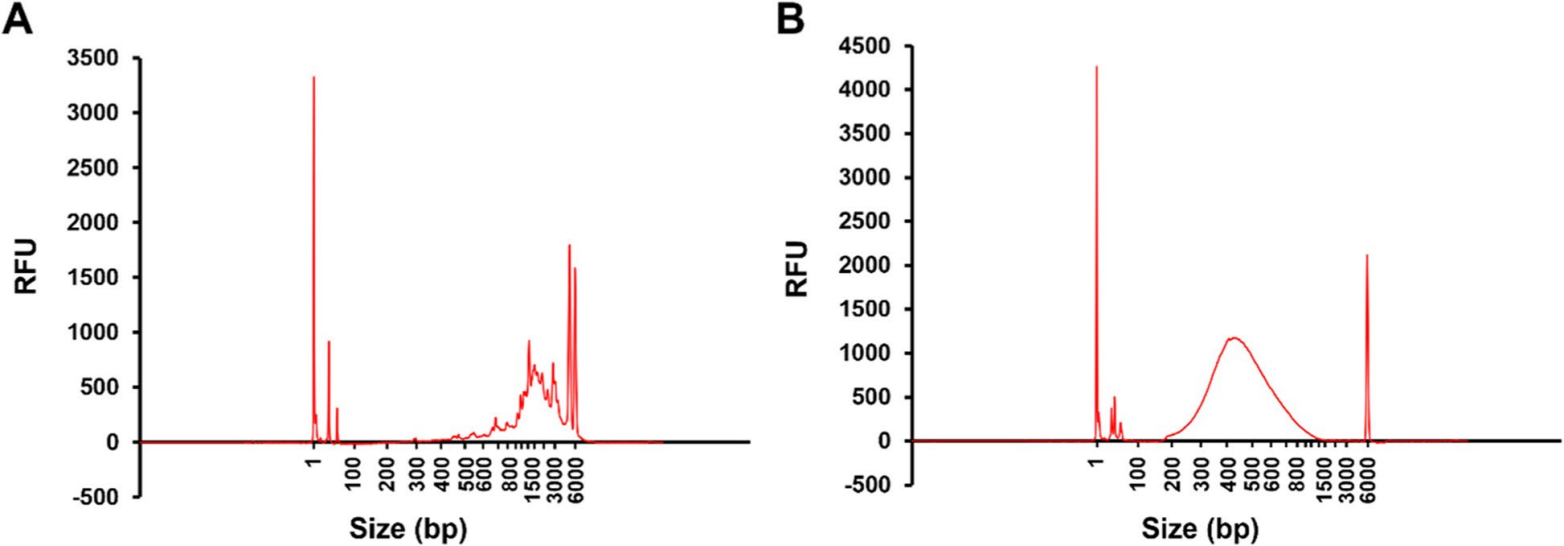
Fragment analyzer electropherograms of representative pre-amplified cDNA and sequencing libraries. **A** Representative profile of a cDNA library synthesized from a single worm’s RNA using the SMART-Seq v4 Ultra Low Input RNA Kit. RFU stands for Relative Fluorescence Units. **B** Representative profile of a sequencing library prepared from a single worm’s cDNA using the Nextera XT DNA Library Prep Kit

With the isolated RNA from single worms as input, we generated cDNA samples using the SMART-Seq v4 Ultra Low Input RNA Kit because of its sensitivity (10 pg–10 ng of total RNA. A single adult worm typically contains ~ 35 ng of total RNA [17]) and its ability to generate full-length cDNAs using template switching technology [11, 18]. For each single-worm sample, after the reverse transcription of mRNA and PCR amplification, the resulting cDNA was of a sufficient amount to perform a quality check using a Fragment Analyzer (FA) (Agilent). The FA results showed a profile of DNA fragments ranging from 500—6,000 bp (Fig. 3A), indicating that full-length cDNAs were generated from full-length mRNA transcripts [14, 15]. If the mRNA transcripts were degraded or insufficiently reverse-transcribed, the cDNA profile would show no peaks or many small peaks [14, 15]. The cDNA samples were then made into sequencing-ready libraries using the Nextera XT DNA Library Prep Kit, which tagments (fragments and tags with adapters) and then amplifies DNA carrying sequencing adapters on both ends. A good sequencing-ready library should have fragment sizes of 250—1,000 bp and a concentration higher than 10 nM, according to the user’s manual of the Nextera XT Kit [19]. Concentration measurements and FA analyses showed that our libraries met these criteria (Fig. 3B and data not shown), indicating successful library preparations from our cDNA samples. These libraries were then pooled and sequenced from both ends (paired-end) on a HiSeq 2500 sequencer. On average, 20 million reads with a length of 2 × 100 bp were generated from each library, which were about half of the number of reads generated for a standard RNA-seq library [16].

### Library quality metrics

**Fig. 4:**
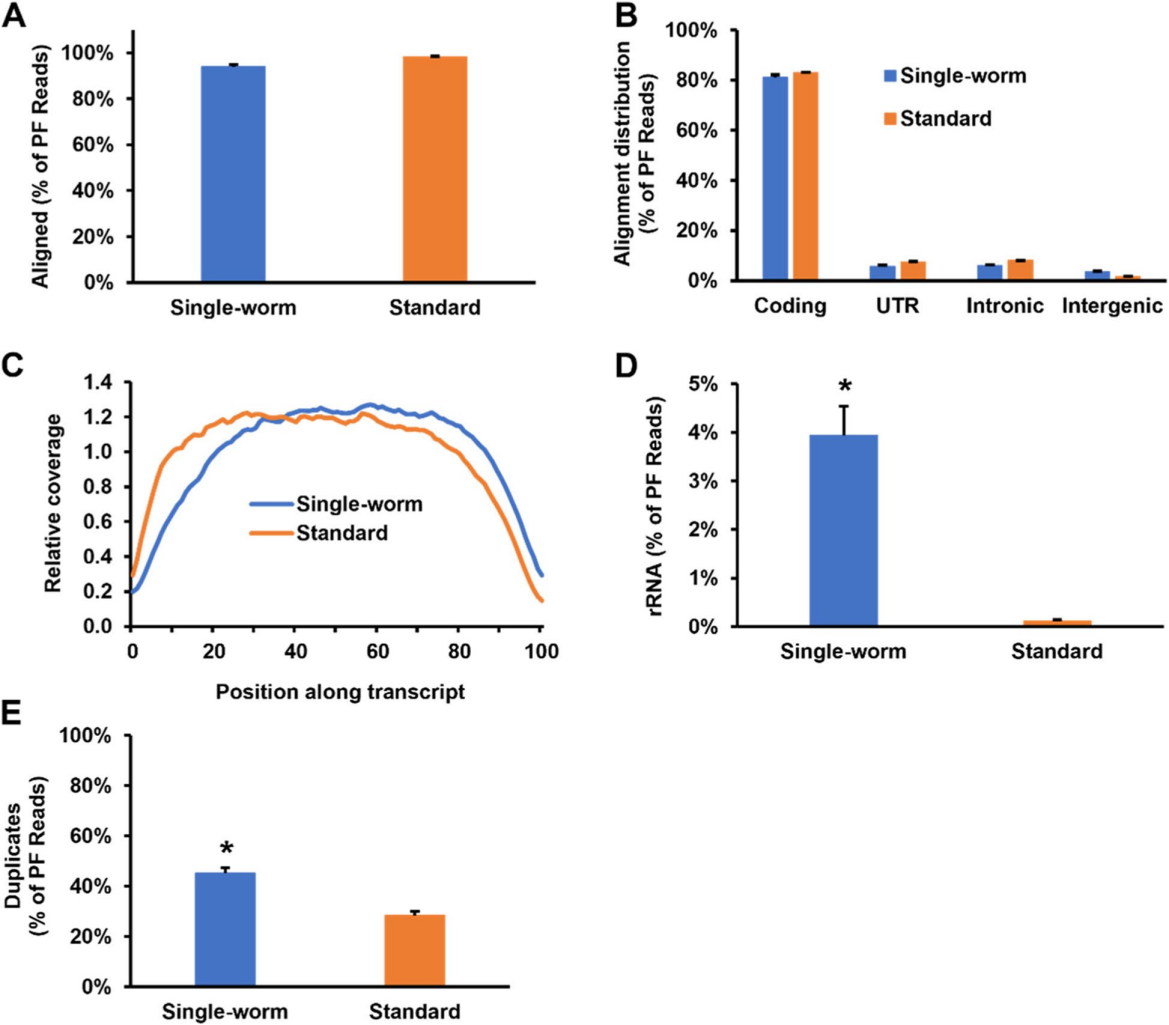
Quality metrics of single-worm and standard RNA libraries. **A** Alignment rates were calculated as the percentage of reads mapped to the *C. elegans* reference genome out of the total passing-filter reads. Each bar in the histogram represents the mean value of five libraries. Error bars represent standard deviation. **B** Alignment distributions of single-worm and standard RNA libraries were calculated as the percentages of reads mapped to the coding, UTR (untranslated region), intronic, and intergenic regions out of the total passing-filter reads. Each bar in the histogram represents the mean value of five libraries. Error bars represent standard deviation. **C** The coverage of various positions of transcripts by mapped reads is shown. Each transcript was subdivided evenly into 1000 bins. Each curve represents the average of five libraries. **D** rRNA content was calculated as the percentage of reads mapped to rRNA sequences out of the total passing-filter reads. Each bar in the histogram represents the mean value of five libraries. Error bars represent standard deviation. The asterisk (*) denotes a significant difference (*p* < 0.05) between the single-worm RNA libraries and the standard RNA libraries. **E** Duplication rates were calculated as the percentage of reads that were duplicates out of the total passing-filter reads. Each bar in the histogram represents the mean value of five libraries. Error bars represent standard deviation. * denotes a significant difference (*p* < 0.05) between the single-worm RNA libraries and the standard RNA libraries

In next-generation sequencing (NGS), a key factor affecting sequence data quality is library quality, i.e., sequence data can only be as good as the input library. Besides the above-mentioned library size and concentration, library quality can be additionally assessed by examining several key parameters, including alignment rate, rRNA content, read coverage over the position of transcripts, and duplicates. Here, we evaluated these parameters in our single-worm RNA-seq libraries and compared them to those observed in standard RNA-seq libraries. To this end, we aligned our sequence data to the *C. elegans* reference genome (ce10, UCSC) using HISAT2 [20] and then analyzed library quality using the Picard tools [21]. On average, 93.84% of the reads from the single-worm libraries were aligned to the reference genome, slightly lower than the alignment rate (98.36%) of the standard libraries (Fig. 4A). Among the aligned reads of the single-worm samples, 81.09% corresponded to coding sequences, 5.57% to untranslated regions (UTRs), 6.05% to intronic sequences, and 3.33% to intergenic sequences, all of which were comparable to the percentages observed in the standard samples (Fig. 4B). While the coverage across transcripts from 5’ to 3’ was balanced in the standard libraries, such coverage in the single-worm libraries was slightly biased towards the 3’ end (Fig. 4C), indicating that the 5’ ends of the RNA molecules in these samples were either not adequately protected from degradation during RNA extraction or were not sufficiently transcribed during cDNA synthesis or both. For rRNA contamination, 3.94% of the reads were rRNA sequences in the single-worm libraries, which was significantly higher than the 0.12% observed in the standard libraries (Fig. 4D). The single-worm libraries also had more duplicates than the standard libraries (45.11% vs. 28.31%) (Fig. 4E). Overall, the quality of the single-worm libraries was comparable to that of the standard libraries, with some parameters slightly worse than the standard. Indeed, by comparison, the quality metrics of our single-worm libraries were better than many of the libraries prepared using other commercial library kits [22, 23].

### Concordance of gene expression quantification

**Fig. 5:**
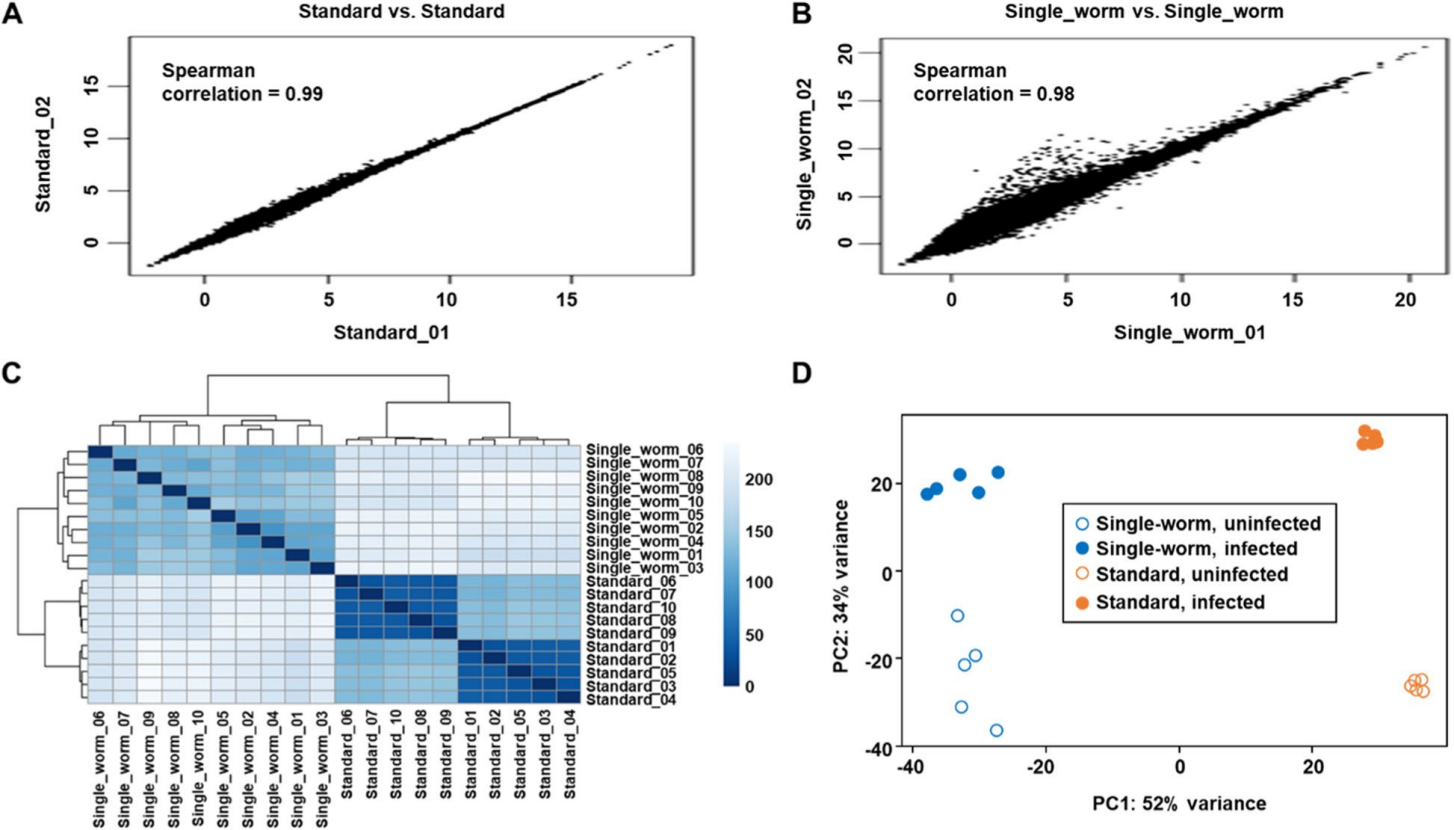
Concordance of expression quantification within single-worm and standard samples. **A** Representative scatter plot of the log_2_(cpm + 1) values of standard libraries (Standard_01 vs. Standard_02) and Spearman’s rank correlation coefficient for the two libraries. **B** Representative scatter plot of the log_2_(cpm + 1) values between single-worm libraries (Single_worm_01 vs. Single_worm_02) and Spearman’s rank correlation coefficient for the two libraries. **C** Heatmap of the sample-to-sample distances of single-worm and standard libraries via hierarchical clustering. Dark blue to light blue represents close to distant clustering. **D** Principal component analysis (PCA) of single-worm and standard libraries using log_2_(cpm + 1) values. Open blue, filled blue, open orange, and filled orange dots represent uninfected single-worm, infected single-worm, uninfected standard, and infected standard libraries, respectively

Consistent quantification of gene expression within biological replicates is the foundation for finding DEGs between different experimental conditions. Here, we evaluated the concordance of expression quantification within single-worm samples as well as within standard samples. Spearman’s rank correlation coefficients between samples based on counts per million (cpm) fragments mapped to exons were used for the concordance evaluations. The correlation coefficients were greater than 0.99 between the standard samples and 0.97—0.98 between the single-worm samples, indicating that the expression quantification was consistent within each of the two methods, with the standard method having slightly better consistency. These results agree with the scatter plots of the log_2_(cpm + 1) values, which showed that all of the standard sample points were aligned along the diagonal (Fig. 5A and Fig. S1A), while some points of the single-worm samples were dispersed (Fig. 5B and Fig. S1B). Furthermore, a heatmap of the sample-to-sample distance matrix demonstrated that the whole transcriptome expression profiles of the standard samples were clustered into infected and uninfected experimental groups, and that the standard samples within both groups were clustered closer to each other than the single-worm samples, respectively (Fig. 5C). Principal component analysis (PCA) recapitulated the hierarchical clustering analysis, with the standard samples being separated from the single-worm samples in the first component, while infected and uninfected samples were separated in the second component (Fig. 5D). In either dimension, the standard samples were closer to each other than the single-worm samples (Fig. 5D). These results indicate that the expression quantification was different between the standard samples and the single-worm samples, possibly due to different library prep protocols and different amounts of input RNA. They also show that there was higher consistency within the standard samples than within the single-worm samples. The reason for this difference could be that the five replicates in the single-worm RNA-seq were five different worms with the same genetic background, whereas the five replicates in the standard RNA-seq were pooled worms that likely masked individual variations.

A source of variance among the single-worm samples could be different efficiency in cell dissociation and/or lysis during the mechanical lysis of worms. To examine this possibility, we compared the expression of tissue-specific genes among the five replicates of single-worm RNA-seq samples. *C. elegans* mainly consists of four tissues, namely, hypodermis, intestine, muscles, and neurons. Kaletsky et al. identified specific genes that are highly expressed and significantly enriched in these tissues, which included 584, 519, 426, and 867 genes in hypodermis, intestine, muscle, and neurons, respectively [24]. Comparisons of our single-worm RNA-seq dataset with these genes revealed that most of these genes (ranging from 89.0 to 99.7%) were detected in each of the five biological replicate samples (Table S2 and Fig. S2A), indicating that the cells in the single-worm samples were well dissociated and lysed during the mechanical lysis step and that the cell dissociation and lysis were consistent among the five biological replicate samples. In addition to comparing the number of tissue-specific genes, we also compared the expression levels of the tissue-specific genes within the single-worm RNA-seq samples. As shown in Fig. S2B, the means of read counts of the tissue-specific genes were comparable among the five replicate samples, supporting the notion that the efficiency of cell dissociation and lysis was consistent among these replicate samples. Additionally, we compared the read counts and variance of expression quantification of the tissue-specific genes between our single-worm RNA-seq and the standard RNA-seq. As shown in Fig. S2C, while the read counts of hypodermis- and intestine-specific genes were comparable between the two methods, the single-worm RNA-seq detected significantly higher expression of muscle-specific genes and the standard RNA-seq detected significantly higher expression of neuron-specific genes. On the other hand, the coefficient of variations (CVs, i.e., standard deviation/mean of read counts) of all tissue-specific genes were higher in the single-worm RNA-seq than those in the standard RNA-seq (Fig. S2D), indicating that the variance of gene expression among the single-worm samples was indeed larger than that among the standard samples. Taken together, these data indicate that the variations across single-worm samples were not originated from inconsistent cell dissociation or lysis but from individual worms’ heterogeneity in gene expression.

Another source of variance between the single-worm samples could be age heterogeneity among synchronized worms. To test this possibility, we measured and compared the expression of vitellogenin genes in single-worm samples using quantitative real-time PCR (qRT-PCR), as vitellogenin expression is tightly regulated and is adult-specific [25]. For these measurements, we used the same RNA samples that were used in our single-worm RNA-seq experiments, so the results should be a true comparison among single-worm RNA-seq samples. Our qRT-PCR results showed that all five single-worm samples had comparable expression of individual vitellogenin genes (i.e., *vit-1*, *vit-2*, *vit-3*,*4*,*5* (detected with the same primers), and *vit-6*) (Fig. S3A). For the purpose of statistical comparison, we normalized the expression of each gene in each sample against sample 1, and then compared the means of the six genes among the different samples (Fig. S3B). Two-sample *t*-tests did not find any significant difference between any two samples, indicating similar expression levels of vitellogenin genes in all five samples (Fig. S3B). These data suggest that all worms in the single-worm RNA-seq experiments were at the adult stage, likely having the same age. Additionally, we also compared the expression levels of vitellogenin genes among the five biological replicate samples in our single-worm RNA-seq data. The read counts of each of the six vitellogenin genes were relatively high and were comparable among the five replicates (Fig. S3C), and the mean read counts of the six genes as a whole were also comparable among the five samples (Fig. S3D), indicating similar expression levels of vitellogenin genes across the replicate samples. These results suggest that the five individual worms used in the single-worm RNA-seq had similar ages, which agrees with the result of the above-described qRT-PCR experiment.

To further investigate the differences in expression quantification between the standard and single-worm samples, we evaluated the concordance of their expression quantification using Spearman rank correlation and scatter plots. Although the quantifications of these two groups of samples appeared to be consistent based on the Spearman rank correlation coefficients (0.96–0.97), a careful examination of their scatter plots revealed that a large number of genes split to the two sides of the diagonal, possibly canceling out the negativity of Spearman rank correlation (Fig. S1C). To find out the identities and functions of these genes, we compared their expression values using DESeq2. Our results showed that among the 24,453 genes detected by both methods, 9,677 genes had significantly higher expression values (1.5-fold or more) in the standard RNA-seq, while 4,863 genes had higher values in the single-worm RNA-seq, which agrees with the scatter plots. Gene ontology (GO) analysis of the higher-value genes in the standard RNA-seq identified 37 significantly enriched biological processes, 33 of which involve signaling (Table S3). This indicates that genes related to signaling were highly expressed in the standard samples compared to the single-worm samples. GO analysis of the higher-value genes in the single-worm RNA-seq identified 556 significantly enriched biological processes, which can be divided into nine categories, namely, metabolic/biosynthetic processes, replication/transcription/translation, organelle organization/localization, reproduction/development, transport, protein modification/activation, stress response, and other processes (Table S4). Among these biological processes, the most enriched was metabolic/biosynthetic processes, followed by reproduction/development and organelle organization/localization, indicating that the single-worm samples had higher metabolism rates and other organismal/cellular activities than the standard samples. This phenomenon could result from bias sampling, that is, healthy worms were chosen for single-worm RNA-seq because they were representative individuals in a population, whereas the population samples inevitably also contained some unhealthy individuals that had lower gene expression in metabolism and other activities. Therefore, gene expression data are the combinational result of animals’ physiological state and how animals are sampled, and both factors should be taken into consideration in data interpretation.

### Differential gene expression in response to *P. aeruginosa* infection

**Fig. 6:**
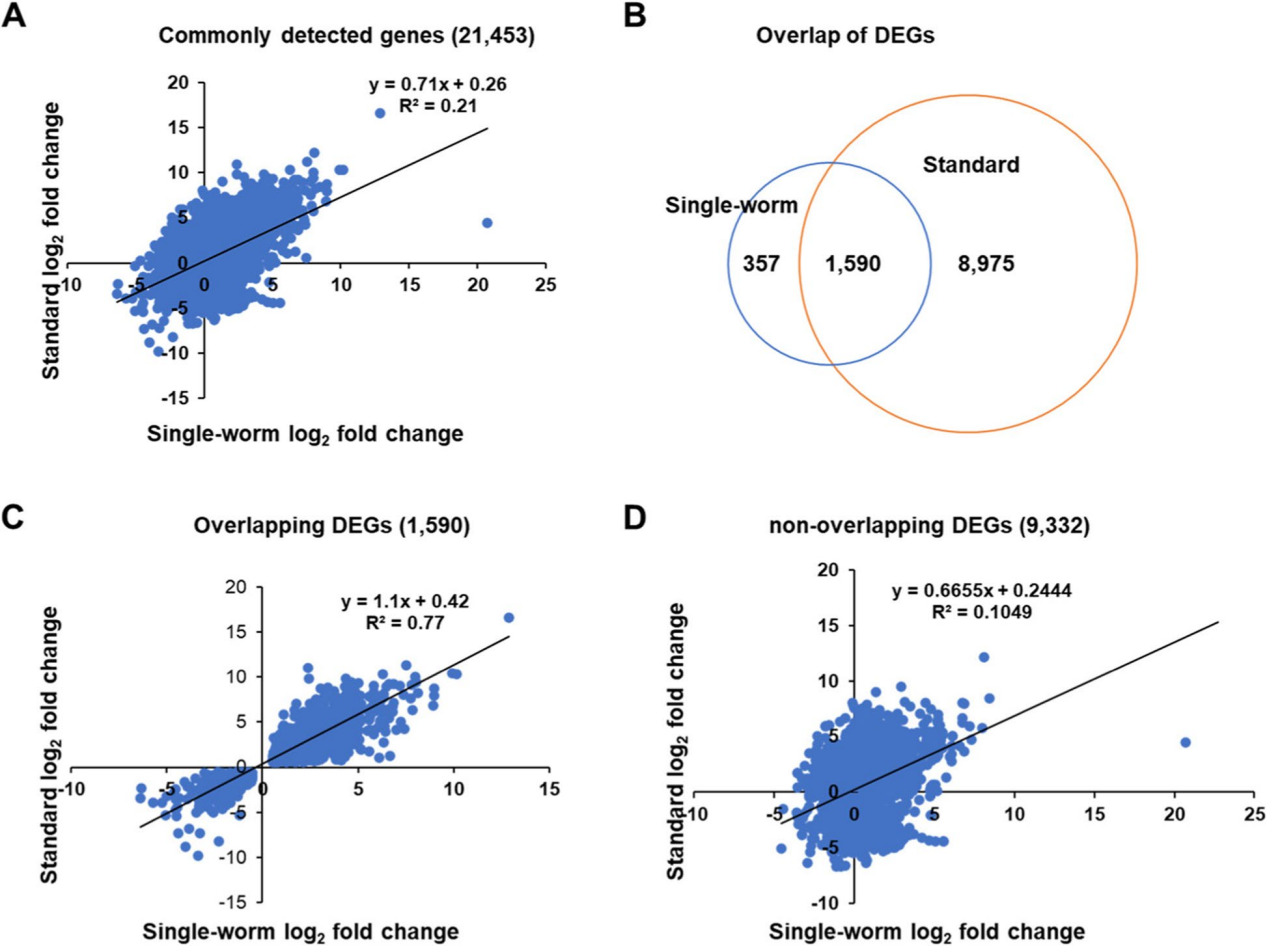
Pathogen infection-induced differential gene expression detected by single-worm and standard RNA-seq. **A** A pairwise scatter plot of the log_2_ ratio values of the 21,453 genes commonly detected by both single-worm and standard RNA-seq. The plot was fitted with a linear regression line, and the equation and R^2^ value are shown. **B** Venn diagram showing the numbers of DEGs significantly changed at least 1.5-fold that were identified by single-worm RNA-seq only, by standard RNA-seq only, and by both methods (overlapping DEGs). **C** A pairwise scatter plot of the log_2_ ratio values of the 1,590 overlapping DEGs. The plot was fitted with a linear regression line, and the equation and R^2^ value are shown. **D** A pairwise scatter plot of the log_2_ ratio values of the 9,332 non-overlapping DEGs. The plot was fitted with a linear regression line, and the equation and R^2^ value are shown

We next assessed how well single-worm RNA-seq could identify DEGs in *C. elegans* upon *P. aeruginosa* infection, compared to the standard RNA-seq method. To this end, five replicates of four group samples (single and bulk worms with or without exposure to *P. aeruginosa* for 24 h) were subjected to RNA-seq analysis. Our results showed that in total, 29,921 genes were identified and quantified with a false discovery rate (FDR) of 5% by the single-worm method, while 22,389 genes were identified by the standard method. The fact that 7,532 fewer genes were identified using the standard method than the single-worm method is surprising given that the RNA input in the single-worm RNA-seq was much lower than that used in the standard RNA-seq (10 ng vs. 2.5 µg), thus the opposite trend was expected. However, the lower RNA input may have brought out the presence of low-copy-number transcripts that was reflected in the sequencing data, indicating that the single-worm method may maintain gene diversity better than the standard method where information regarding low-copy-number transcripts could possibly be lost or diminished due to the presence of more high-copy-number transcripts. A pairwise scatter plot of log_2_ ratio values of differential gene expression indicates that the overall differential expression measured by these two methods had little correlation with each other (R^2^ for linear regression = 0.21) (Fig. 6A). When the adjusted *p-value* was set at < 0.05 to examine the differential gene expression between infected and control samples, only 1,947 genes were significantly regulated (upregulated or downregulated) at least 1.5-fold in the single-worm RNA-seq, whereas 10,565 genes were significantly regulated at least 1.5-fold in the standard RNA-seq, four times higher than the number in the former. These data suggest that the genes found by the single-worm method were indeed diversified among the 10 individual worms (5 infected worms and 5 uninfected control worms), and that the standard method had greater power to detect DEGs. An unsupervised hierarchical clustering of the DEGs detected in the standard RNA-seq showed that the genes were similarly up- or down-regulated upon pathogen infection in all five biological replicates of the standard samples, indicating great uniformity across the standard samples (Fig. S4). In the single-worm samples, however, most of these genes were not significantly regulated, and the samples exhibited largely similar but distinct patterns of gene expression (Fig. S4), indicating that the gene expression was diversified among the individual worms and that gene regulation could occur at the individual level. Among the 1,947 DEGs found by the single-worm method, most (1,590 or 82%) were also detected by the standard method (Fig. 6B). Fitting a pairwise scatter plot of the differential expression values of the common DEGs using the linear regression method yielded an R^2^ of 0.77 (Fig. 6C), indicating a moderate correlation between the expression of DEGs measured by the two methods. However, a similar fitting of the scatter plot between the non-overlapping DEGs of the two methods resulted in an R^2^ of 0.10 (Fig. 6D), indicating little correlation between the non-overlapping DEGs. Taken together, these data suggest that the gene regulation revealed by the single-worm method was specific and could be an essential part of the overall gene regulation uncovered by the standard method.

To further investigate why the single-worm RNA-seq failed to detect a large number of DEGs that were detected in the standard RNA-seq, we performed data analysis at two levels. At the first level, we compared the read counts and variance of expression quantification between fail-to-detect DEGs and DEGs in the single-worm RNA-seq. At the second level, we compared the read counts and variance of expression quantification of fail-to-detect DEGs between the single-worm and standard RNA-seq. In the single-worm RNA-seq, the average read counts of DEGs were higher than those of fail-to-detect DEGs (Fig. S5A), while the CV of the former was lower than that of the latter (Fig. S5B), suggesting that both low expression values and high worm-to-worm variations of some genes were likely responsible for the failure of their detection as DEGs in the single-worm RNA-seq. At the second level, the fail-to-detect DEGs in the single-worm RNA-seq had significantly lower read counts but significantly higher CV than those in the standard RNA-seq (Fig. S5, C and D). Taken together, these data support the notion that both low read counts and large variations between the single-worm samples could contribute to the failure of detection of many DEGs in the single-worm RNA-seq.

**Fig. 7:**
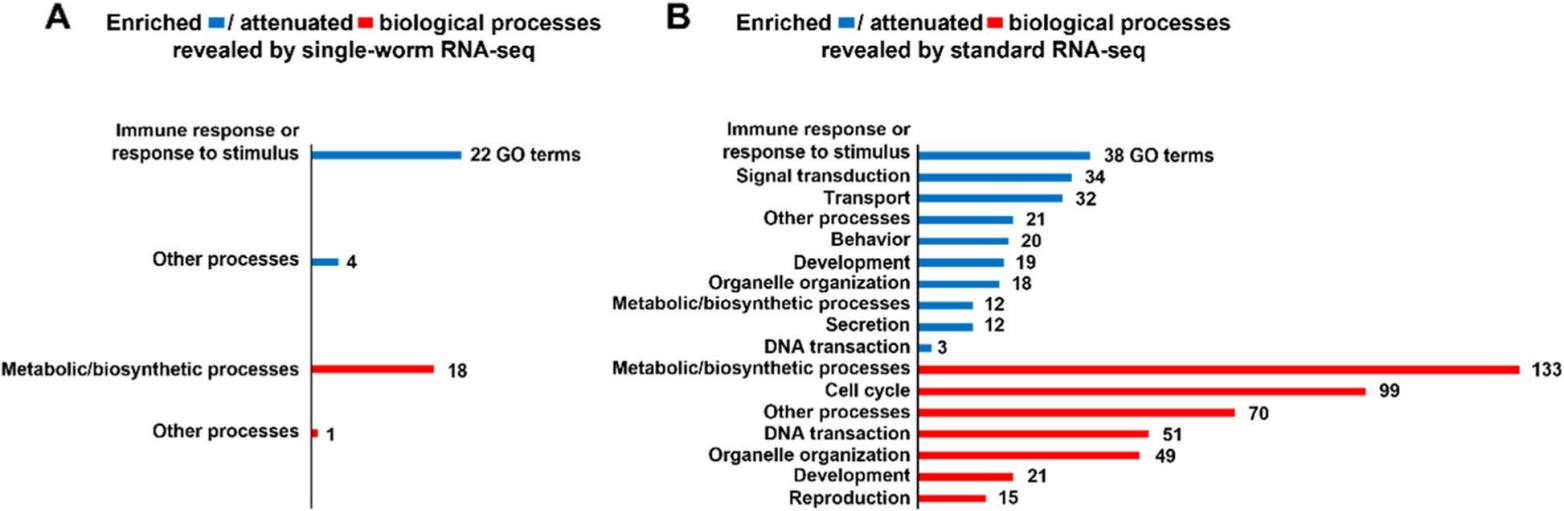
Gene ontology (GO) analysis of pathogen infection-induced DEGs identified by single-worm and standard RNA-seq. Pathogen infection-induced DEGs identified by single worm RNA-seq (**A**) and standard RNA-seq (**B**) were subjected to GO analysis. Blue bars represent enriched biological processes resulted from DEGs that were significantly upregulated at least 1.5-fold with pathogen infection. Red bars represent attenuated biological processes resulted from DEGs that were significantly downregulated at least 1.5-fold with pathogen infection. In each panel, the length of the bar corresponds to the number of GO terms in a specific GO category

We next investigated the biology of the gene regulation revealed by the two methods. Among the significantly regulated genes in the single-worm RNA-seq, 1,385 genes were up-regulated and 562 genes were down-regulated at least 1.5-fold in infected worms relative to uninfected controls. GO analysis of the 1,385 up-regulated genes identified 26 significantly enriched biological processes, 22 of which involve the immune response or response to stimulus (Fig. 7A and Table S5). This is consistent with the fact that *P. aeruginosa* is pathogenic to *C. elegans* and can trigger a strong immune response in the worm [26–29]. GO analysis of the 562 down-regulated genes identified 19 significantly attenuated biological processes, 18 of which involve metabolic/biosynthetic processes (Fig. 7A and Table S6). This indicates the likely alteration of metabolism in the worm whilst fighting off the infection [30]. Among the significantly regulated genes in the standard RNA-seq, 6,095 genes were up-regulated and 4,470 genes were down-regulated at least 1.5-fold in infected worms relative to uninfected controls. GO analysis of the 6,095 up-regulated genes identified 209 significantly enriched biological processes, 38 of which involve the immune response or response to stimulus, the most among all of the categories of enriched GO terms (Fig. 7B and Table S7). The same analysis of the 4,470 down-regulated genes identified 438 significantly attenuated biological processes, 133 of which involve metabolic/biosynthetic processes, the most among all of the categories of attenuated GO terms (Fig. 7B and Table S8). A comparison of the infection-affected biological processes revealed by the two methods (Fig. 7A vs. B) suggests that the single-worm method captured the core biological processes affected by infection, i.e., the immune response or the response to stimulus was upregulated, while metabolic/biosynthetic processes were downregulated. This is consistent with the observation that most DEGs identified by the single-worm method were also detected by the standard method (Fig. 6B), indicating that the single-worm method detected the core set of DEGs influenced by *P. aeruginosa* infection. Taken together, our results suggest that the single-worm method could be used to identify commonly regulated genes among individual worms and such gene regulation may mediate a population’s core physiological responses to endogenous or environmental stimuli.

## Discussion

In this study, we developed a single-worm RNA-seq method to profile gene expression in individual *C. elegans* and evaluated its performance and application in library preparation and DEG detection by comparing it to a standard RNA-seq method. Overall, the key aspects of our single-worm RNA-seq protocol, including library and sequence data quality, are comparable to what is seen with standard RNA-seq. When used for the analysis of gene differential expression induced by pathogen infection, the single-worm RNA-seq method identified the core set of DEGs and biological processes affected by infection. These results demonstrate the effectiveness of our single-worm RNA-seq method in transcriptome profiling and its usefulness in addressing biological questions.

Previously, a similar single-worm RNA-seq method was developed by Dillman and colleagues [14, 15], in which the worms were lysed chemically and the RNA was reverse transcribed into cDNA using the SMART-Seq2 reagents. In our study, we found that harsh chemical lysis conditions, which are required for penetrating the worm’s thick cuticle, contributed to RNA degradation, and that mechanical lysis of *C. elegans* using a TissueLyser maintained RNA integrity (Fig. 2). This suggests that mechanical lysis of worms can overcome a major technical challenge in *C. elegans* research, namely, breaking the worm’s cuticle without degrading macromolecules. The nematode cuticle is a highly structured extra-cellular matrix comprised predominantly of cross-linked collagens, making *C. elegans* resilient to buffer extraction [31]. Buffers with high concentrations of detergent and/or proteinase K that lyse the cuticle also tend to degrade macromolecules and inactivate enzymes. Indeed, a major obstacle for biochemical studies using *C. elegans* is the high difficulty of obtaining functionally active nuclear extracts due to the nematode’s thick surrounding cuticle [32]. Recent studies have shown that use of a Balch homogenizer to mechanically disrupt worms yields functional protein extracts [32, 33], and that nuclear extracts prepared using this method could be used to reconstitute in vitro transcription reactions [32]. These studies, along with ours, demonstrate that the mechanical lysis of worms using devices such as a TissueLyser or a Balch homogenizer is a good way to overcome the cuticle issue for nucleic acid isolation or nuclear extract preparation and should be promoted for wide use in biochemical studies in *C. elegans*.

In the current study, we systemically evaluated our single-worm RNA-seq method by assessing library quality, concordance of gene expression quantification, and the ability to detect DEGs. Overall, the quality of our single-worm libraries was comparable to that of the standard libraries. However, the concordance of gene expression quantification within the single-worm samples was less consistent than that seen within the standard samples. The reason for this difference could be that the five replicates in the single-worm RNA-seq were five different worms with the same genetic background, whereas the five replicates in the standard RNA-seq were worm pools that likely masked individual variations. This notion was supported by our analysis of pathogen infection-induced differential gene expression. While the single-worm method captured the core set of DEGs and biological processes affected by infection, many DEGs and regulated biological processes identified by the standard method were not detected by the single-worm method (Figs. 6 and 7), indicating that there was gene regulation that occurred in some worms but was not shared by all individuals. For example, signal transduction was the second most enriched GO term category (34 GO terms) detected by the standard RNA-seq, but no such GO terms were enriched in the single-worm RNA-seq (Fig. 7), indicating that signal transduction was not a shared response to pathogen infection but only occurred in some individuals. Since neural signaling is known for regulating the intensity of the immune response [34], it could explain why individual worms with the same genetic background may exhibit different immunities, mirrored by differences in their survival rates (ranging from one to four days) against *P. aeruginosa* infection [30, 35]. Our study demonstrates that combing the single-worm and standard RNA-seq approaches can detect and distinguish shared and individual-specific gene activities in worm populations. Further development and application of single-worm RNA-seq will certainly help solve the mystery of individual heterogeneity in isogenic populations.

## Conclusions

In this study, we have developed a single-worm RNA-seq method to effectively profile gene expression in individual *C. elegans* and have applied this method to study *C. elegans* responses to pathogen infection. The key aspects of our single-worm RNA-seq protocol, including the integrity of the starting RNA as well as the quality of the library and sequence data, are comparable to standard RNA-seq. However, the concordance of gene expression quantification within the single-worm samples was less consistent than that observed within the standard samples, likely because the replicates in the single-worm RNA-seq were different individual worms with the same genetic background, while the replicates in the standard RNA-seq were worm pools that likely masked individual differences. When applied to examine pathogen-induced differential gene expression, the single-worm method captured the core set of DEGs and biological processes affected by infection. However, many DEGs and regulated biological processes identified by the standard method were not detected by the single-worm method, indicating that there was gene regulation that occurred at the level of individual worms but was not shared by all individuals. Our study demonstrates that by using both the single-worm and standard RNA-seq approaches, we can detect and distinguish both shared and individual-specific gene activities in a worm population. Thus, furthering the development and application of single-worm RNA-seq will certainly contribute to addressing the heterogeneity issue of individuals with the same genetic background.

## Methods

### *C. elegans*, bacterial strains, and infection

The wild-type *C. elegans* Bristol N2 strain was used in this study. Worms were cultured under standard conditions and fed *Escherichia coli* OP50 [36]. *E. coli* OP50 and the pathogen *P. aeruginosa* PA14 were grown in Luria–Bertani broth at 37 °C. Worm infection with *P. aeruginosa* was done as previously described [16]. Briefly, worms were synchronized using the bleaching method following our previously published protocol [16]. Synchronized L1 larval animals were grown to L4 stage on *E. coli OP50* at 20 °C, then transferred to NGM plates containing *P. aeruginosa* PA14 and incubated at 25 °C for 24 h. Uninfected control worms went through the same process with *E. coli* OP50 in place of *P. aeruginosa* PA14. The animals were then collected and washed with M9 buffer for downstream use.

### Testing conditions for worm lysis and RNA extraction

Three conditions for worm lysis were tested followed by RNA extraction.

Condition 1 – Thirty worms per group were disrupted and homogenized using a TissueLyser LT (Qiagen) following the “Purification of RNA or Multiple Analytes from Animal and Human Tissues” protocol in the manufacturer’s manual. Briefly, worms immersed in 30 µL of RNALater solution were transferred to a 2 mL microcentrifuge tube that was precooled with dry ice and contained one stainless steel bead (5 mm mean diameter). The sample was then incubated on dry ice for 15 min and placed into the insert of the TissueLyser LT Adapter. After incubation at room temperature for 2 min, 600 µL of Buffer RLT from the RNeasy Mini Kit were added. The worms were then disrupted in a TissueLyser LT for 4 min at 50 Hz. The resulting lysate was immediately subjected to RNA purification using the RNeasy Mini Kit following the “Purification of Total RNA from Animal Tissues” protocol in the manufacturer’s handbook. Briefly, the lysate was centrifuged at 16,873 × *g* for 3 min. The supernatant was carefully transferred to a new microcentrifuge tube, and one volume of 70% ethanol was added to the supernatant. The mixture was then applied to an RNeasy spin column, followed by one wash with Buffer RW1 and two washes with Buffer RPE. Finally, the RNA was eluted with 30 µL of RNase-free water.

Condition 2 – Thirty worms were resuspended in 30 µL of Lysis Buffer (50 mM KCl, 10 mM Tris–HCl, pH 8.3, 2.5 mM MgCl_2_, 0.45% Triton X-100, 0.45% Tween-20, 0.11% gelatin (w/v), 100 µg/mL proteinase K, 200 units of RNasin Ribonuclease Inhibitor), heated at 65 °C for 10 min, followed by 1 min at 85 °C, and then held at 4 °C in a thermocycler. The worms were then transferred to a 2 mL microcentrifuge tube that was precooled with dry ice and contained one stainless steel bead. Homogenization using a TissueLyser LT and RNA extraction using the RNeasy Mini Kit were performed as described in Condition 1.

Condition 3 – Thirty worms were resuspended in 30 µL of Lysis Buffer, heated at 65 °C for 10 min, followed by 1 min at 85 °C, and then held at 4 °C in a thermocycler. The worms were then disrupted and homogenized using a QIAshredder spin column (Qiagen). Briefly, 600 µL of Buffer RLT were added to the worms, and then they were transferred to a QIAshredder spin column with a 2 mL collection tube. The column was centrifuged at 16,873 × *g* for 2 min, and the flowthrough was used for RNA extraction using the RNeasy Mini Kit, as described in Condition 1.

Following extraction, the resulting RNA samples were subjected to concentration measurements using a Qubit fluorometer with the RNA HS assay Kit (Life Technologies) as well as integrity checks using a Fragment Analyzer with the DNF-472 RNA Kit (15 nt) (Agilent).

### Single-worm RNA-seq

Our single-worm RNA-seq workflow includes five steps: worm lysis and RNA extraction, cDNA synthesis, library preparation, sequencing, and sequence data analysis. Below is a description of these steps.

One worm per group immersed in 10 µL of RNALater solution was disrupted and homogenized using a TissueLyser LT under the conditions described in Condition 1 above. The isolated RNA was used for cDNA synthesis using the Takara SMART-Seq v4 Ultra Low Input RNA Kit per the manufacturer’s protocol. Briefly, first-strand cDNA was synthesized from 9.5 µL of extracted RNA. The cDNA was then amplified using SeqAmp DNA polymerase by running the LD PCR program (95 °C for 1 min; 12 cycles of 98 °C for 10 s, 65 °C for 30 s, 68 °C for 3 min; 72 °C for 10 min; 4 °C forever). The amplified cDNA was purified using AMPure XP beads (Beckman Coulter) and resuspended in 15 µL of Elution Buffer. Finally, 2 µL of cDNA was aliquoted for validation using a Fragment Analyzer with the High Sensitivity NGS Fragment Analysis Kit (Agilent), and the remaining cDNA was used for sequencing library preparation.

A sequencing library was generated for each sample using the Nextera XT DNA Library Preparation Kit (Illumina). Briefly, 200 pg of cDNA was tagmented and then amplified using a limited-cycle PCR program per the manufacturer’s instructions. The resulting library was purified using AMPure XP beads and resuspended in 50 µL of Resuspension Buffer (RSB, 10 mM Tris–HCl, pH8.5). The library was then subjected to concentration measurements using a StepOnePlus Real-Time PCR System (ThermoFisher Scientific) along with the KAPA Library Quantification Kit (Kapabiosystems). A quality check was also performed using a Fragment Analyzer with the High Sensitivity NGS Fragment Analysis Kit.

Sequencing was done on a HiSeq2500 sequencer (Illumina) as previously described [16]. Briefly, libraries were diluted to 4 nM with RSB, pooled, and denatured with 0.1 N NaOH. Twenty pM libraries were clustered onto a high-output flow cell in a cBot (Illumina) and sequenced on a HiSeq 2500 from both ends (paired-end) with a read length of 100 bp, per the manufacturer’s instructions. The raw BCL files were converted to FASTQ files using the software program bcl2fastq2.17.1.14. Adaptors were trimmed from the FASTQ files during the conversion. On average, 20 million reads of 2 × 100 bp were generated for each sample.

Sequence data (FASTQ files) were aligned to the *C. elegans* reference genome (ce10, UCSC) using HISAT2 (version 2.2.1) [20]. Library quality metrics were assessed using the Picard tools (version 2.26.5) (CollectRnaSeqMetrics and MarkDuplicates) [21]. Gene expression quantification and differential expression were analyzed using featureCounts (part of the Subread package, version 2.0.3) [37] and DESeq2 (version 1.36.0) [38], respectively. The sequencing data (FASTQ files) were deposited in the NCBI’s Sequence Read Archive (SRA) database through the Gene Expression Omnibus (GEO), and the processed gene quantification files and differential expression files were deposited in the GEO. The complete dataset can be accessed through the GEO with the accession number GSE197834. To evaluate the concordance of gene expression quantification, we generated a heatmap of the sample-to-sample distances and a PCA plot of the samples using the heatmap and plotPCA functions in DESeq2, respectively [39]. An unsupervised hierarchical clustering of the DEGs detected in the standard RNA-seq was performed using ComplexHeatmap [40] following the protocol of Lewis et al. [41]; the clustering was done based on the read counts of each gene in both the standard and the single-worm RNA-seq. Gene ontology (GO) enrichment analyses were conducted to identify significantly regulated biological processes using the web-based program Gorilla (http://cbl-gorilla.cs.technion.ac.il/) [42].

### qRT-PCR

Total RNA was obtained as described above and subjected to qRT-PCR following our published protocol with modifications [30]. Briefly, reverse transcription was done using the SMART-Seq v4 Ultra Low Input RNA Kit (Takara Bio). Quantitative PCR was conducted on StepOnePlus Real-Time PCR system using Power SYBR Green PCR Master Mix in a 96-well plate format (Applied Biosystems). Relative fold changes for transcripts were calculated using the comparative *C*_T_ (2^−ΔΔC^T) method and normalized to pan-actin (*act-1*, *act-3*, and *act-4*). Cycle thresholds of amplification were determined by StepOne Software v2.3 (Applied Biosystems). All samples were run in triplicate. Primer sequences for *vit-1*, *vit-2*, *vit-3*,*4*,*5*, and *vit-6* were described in Dowen et al. [43] and are also available upon request.

### Standard RNA-seq

Standard RNA-seq was done in the study of Sellegounder et al. [16]. Sequence data (FASTQ files) were retrieved from the NCBI’s SRA database through the GEO using the accession number GSE122544. Below is a brief description of the standard RNA-seq protocol. RNA was extracted from five replicates of *P. aeruginosa*-infected or uninfected control worms using QIAzol lysis reagent (Qiagen) and purified with the RNeasy Plus Universal Kit (Qiagen). RNA samples (2.5 µg per sample) with RQNs ranging from 8 to 10 were used for sequencing library preparation using the TruSeq Stranded mRNA Library Prep Kit (Illumina). Libraries were quantified, pooled, denatured, and sequenced on a HiSeq 2500 sequencer. On average, 40 million reads with a read length of 2 × 100 bp were generated for each sample. Sequence data (FASTQ files) were subjected to bioinformatics analysis as described above for the single-worm RNA-seq.

### Statistical analysis

The two-sample *t* test was conducted for the pairwise comparisons shown in Figs. 2A, B and 4A, B, D, E, S2B, C, D, S3A, B, D, and S5; *p* values < 0.05 were considered significant [16].

## Supplementary Information

**Additional file1: Fig. S1-S5.**

**Additional file 2: Table S1.** Five biological replicates of four group samples used in this study.

**Additional file 3: Table S2.** Tissue-specific genes detected in single-worm samples.

**Additional file 4: Table S3.** Enriched biological processes revealed by standard RNA-seq relative to single-worm RNA-seq.

**Additional file 5: Table S4.** Enriched biological processes revealed by single-worm RNA-seq relative to standard RNA-seq.

**Additional file 6: Table S5.** Enriched biological processes revealed by single-worm RNA-seq (infected samples relative to uninfected samples).

**Additional file 7: Table S6.** Attenuated biological processes revealed by single-worm RNA-seq (infected samples relative to uninfected samples).

**Additional file 8: Table S7.** Enriched biological processes revealed by standard RNA-seq (infected samples relative to uninfected samples).

**Additional file 9: Table S8.** Attenuated biological processes revealed by standard RNA-seq (infected samples relative to uninfected samples).

## Data Availability

The RNA sequencing data have been deposited in the NCBI’s Sequence Read Archive (SRA) database through the Gene Expression Omnibus (GEO). The processed gene quantification files and differential expression files have also been deposited in the GEO. The complete dataset can be accessed through the GEO with the accession number GSE197834.

## Abbreviations

RNA-seq: RNA sequencing
NGS: Next-generation sequencing
DEG: Differentially expressed gene
RQN: RNA quality number
FA: Fragment Analyzer
UCSC: University of California, Santa Cruz
UTR: Untranslated region
rRNA: Ribosomal RNA
PCA: Principal component analysis
FDR: False discovery rate
GO: Gene ontology
NCBI: National Center for Biotechnology Information
SRA: Sequence Read Archive
GEO: Gene Expression Omnibus
