# Supplementary material for: Using single-worm RNA sequencing to study C. elegans responses to pathogen infection
Source: BMC Genomics. 2022 Sep 14;23:653. doi: 10.1186/s12864-022-08878-x (PMC9472404; doi:10.1186/s12864-022-08878-x)
Supplement: Supplementary file 1 — Additional file1: Fig. S1-S5. [file 12864_2022_8878_MOESM1_ESM.pdf]

Figure S1

A Standard vs. Standard

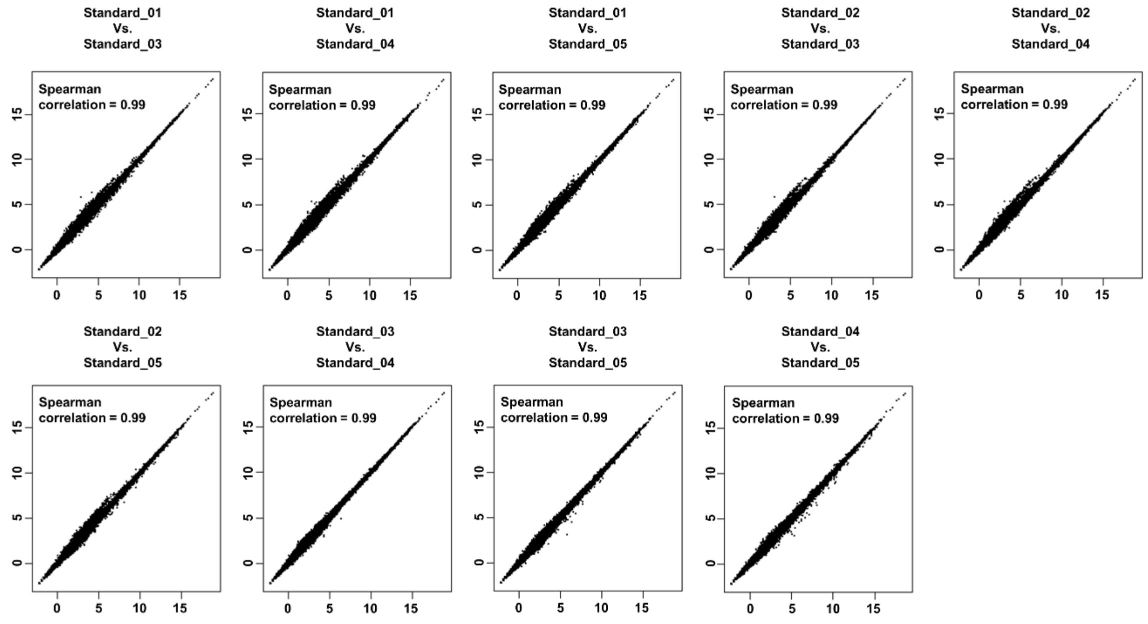

B Single_worm vs. Single_worm

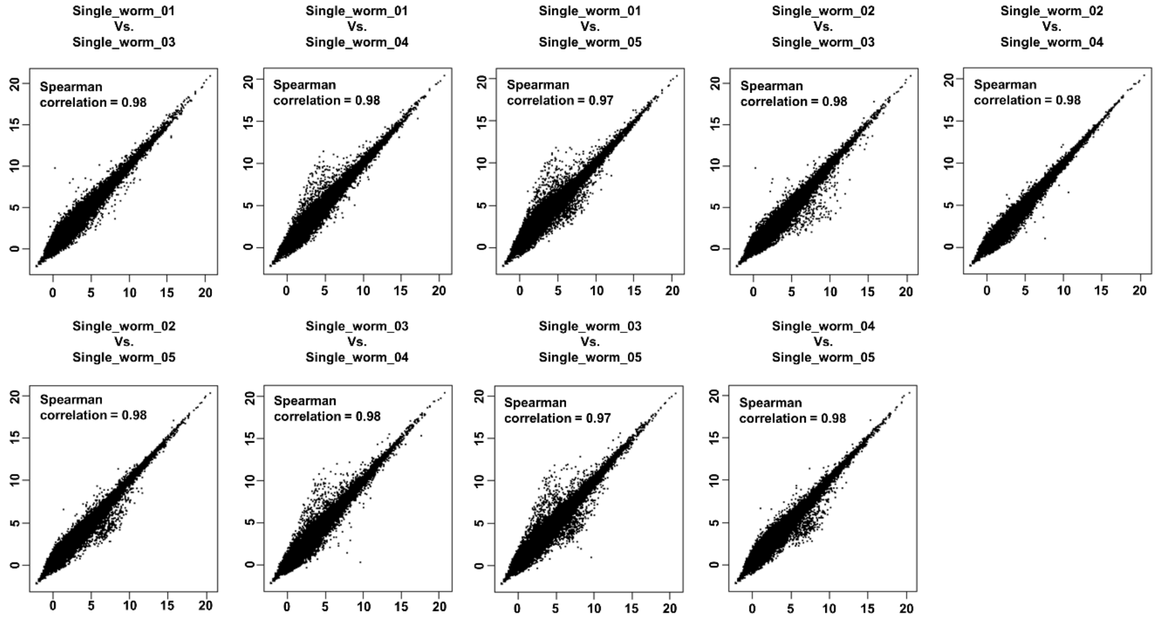

C Single_worm vs. Standard

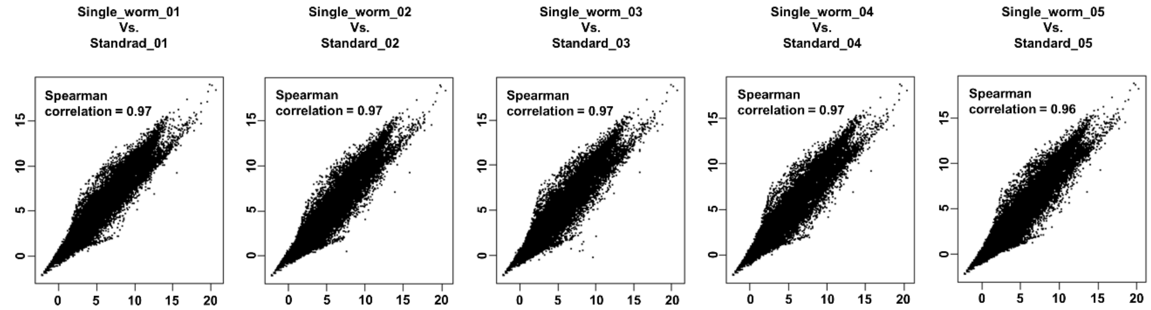

Fig. S1. Scatter plot of the log2(cpm+1) values and Spearman's rank correlation coefficient between samples within the standard RNA-seq (A), between samples within the single-worm RNA-seq (B), as well as between the single-worm and standard samples (C).

Figure S2

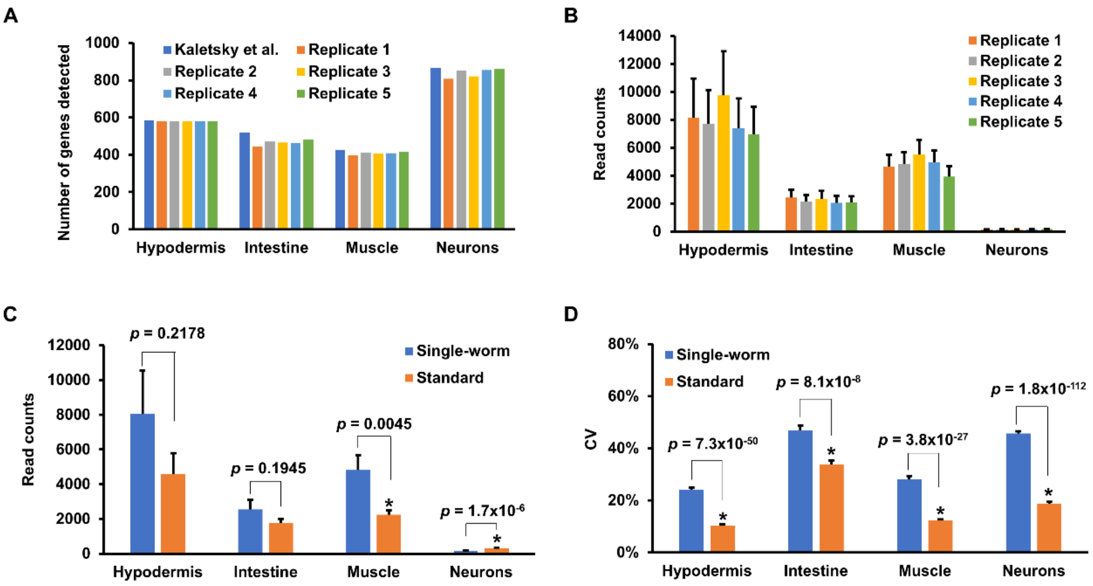

Fig. S2. Tissue-specific gene expression detected by the single-worm and standard RNA-seq. (A) The tissue-specific genes identified by Kaletsky et al. (2018 PLoS Genetics 14(8): e1007559) were used as a reference. The numbers of such tissue-specific genes found in each of the single-worm samples were plotted and compared to the reference. **(B)** The mean read counts of tissue-specific genes in each of the single-worm samples were plotted. The values were compared between samples within each tissue category using the two-sample *t* test, and no significant difference was found between any two samples. Error bars represent standard deviation. **(C)** The mean read counts of tissue-specific genes were compared between the single-worm and standard samples. Error bars represent standard deviation. The asterisk (*) denotes a significant difference between the single-worm and the standard samples. **(D)** The coefficient of variations (CVs) of read counts of tissue-specific genes were compared between the single-worm and standard samples. Error bars represent standard deviation. * denotes a significant difference between the single-worm and the standard samples.

Figure S3

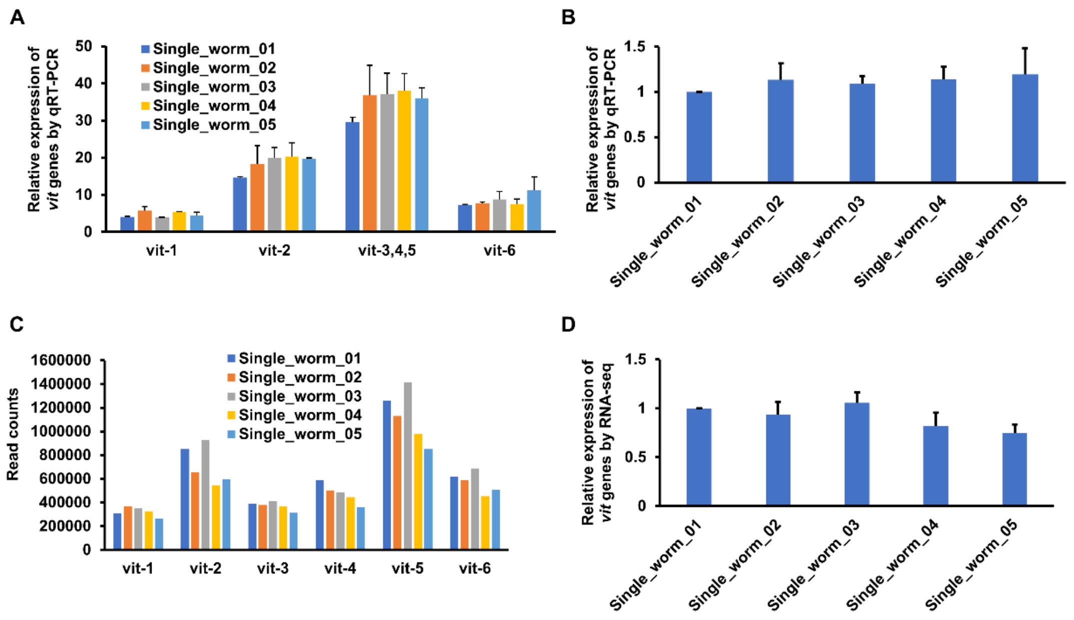

Fig. S3. The expression levels of vitellogenin genes in the five biological replicate samples of the single-worm RNA-seq. (A) qRT-PCR was performed to measure the expression of *vit* genes relative to pan-actin in the five single-worm samples. Error bars represent standard deviation of three technical replicates. **(B)** The means of relative expression of six *vit* genes in the five samples measured by qRT-PCR. The expression value of each *vit* gene in the five replicates were normalized against replicate 1. Columns represent the means of the relative expression of the six *vit* genes. Error bars represent standard deviation. Pairwise comparisons between the five replicates were done using the two-sample *t* test, and no significant difference was found between any two samples. **(C)** Read counts of six *vit* genes in the five replicates measured by the single-worm RNA-seq. **(D)** The means of relative expression of six *vit* genes in the five replicates measured by the single-worm RNA-seq. The read counts of each *vi*t gene in the five replicates were normalized against replicate 1. Columns represent the means of the relative expression of the six *vit* genes. Error bars represent standard deviation. Pairwise comparisons between the five replicates were done using the two-sample *t* test, and no significant difference was found between any two samples.

Figure S4

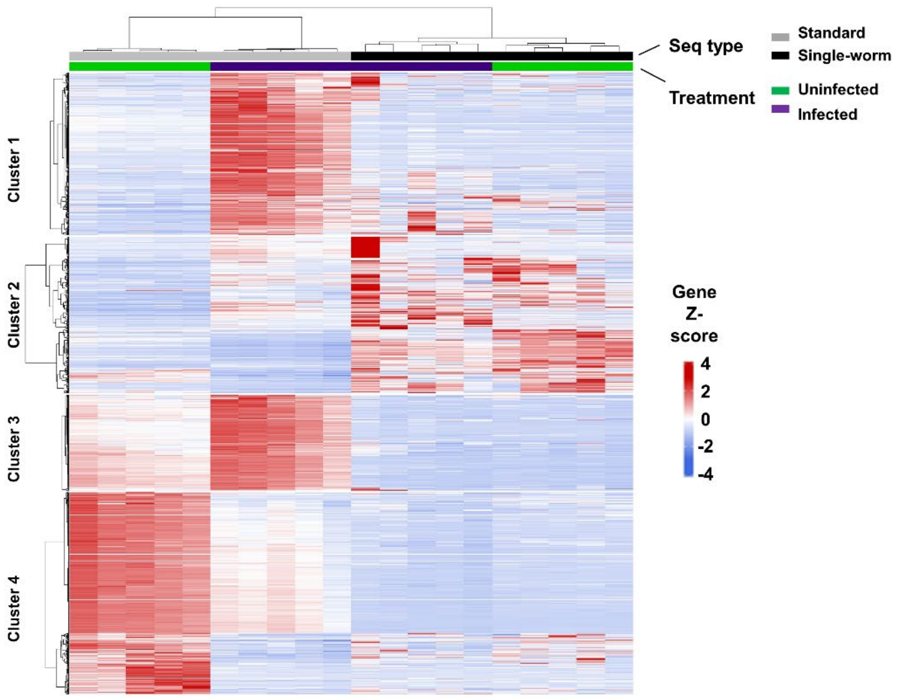

Fig. S4. Clustering analysis of the DEGs detected in the standard RNA-seq. Unsupervised hierarchical clustering was done based on the read counts of each gene in both the standard and the single-worm RNA-seq using ComplexHeatmap. The resulting heatmap showed that the genes in the standard RNA-seq were clustered into four clusters, while these genes in the single-worm RNA-seq were largely not significantly regulated and exhibited largely similar but distinct patterns of expression among the five single-worm samples.

Figure S5

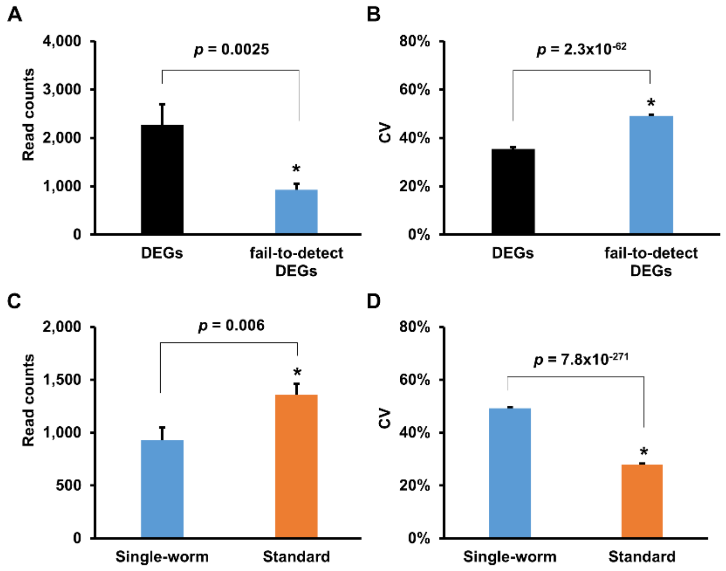

Fig. S5. Read counts and variance of expression quantification of DEGs and fail-to-detect DEGs. (A) The means of read counts of DEGs (black column) and fail-to-detect DEGs (blue column) in the single-worm RNA-seq were calculated by averaging the read counts of all genes in each of these two groups. Error bars represent Standard Error of the Mean (SEM). The asterisk (*) denotes a significant difference between DEGs and fail-to-detect DEGs. *p* value was calculated using the two-sample *t*-test assuming unequal variances. **(B)** Coefficient of variation (CV) was calculated by averaging the CVs of all genes in the DEG group (black column) or the fail-to-detect DEG group (blue column) in the single-worm RNA-seq. Each gene's CV was calculated as standard deviation of read count divided by mean of read count. Error bars represent SEM. * denotes a significant difference between DEGs and fail-to-detect DEGs. *p* value was calculated using the two-sample *t*-test assuming unequal variances. **(C)** The means of read counts of fail-to-detect DEGs in the single-worm (blue column) and standard (orange column) RNA-seq was calculated as in (A). Error bars represent SEM. * denotes a significant difference between single-worm and standard RNA-seq. *p* value was calculated using the two-sample *t*-test assuming unequal variances. **(D)** The CVs of fail-to-detect DEGs in the single-worm (blue column) and standard (orange column) RNA-seq were calculated as in (B). Error bars represent SEM. * denotes a significant difference between single-worm and standard RNA-seq. *p* value was calculated using the two-sample t-test assuming unequal variances.
