# Supplementary material for: Using single-worm RNA sequencing to study C. elegans responses to pathogen infection
Source: BMC Genomics. 2022 Sep 14;23:653. doi: 10.1186/s12864-022-08878-x (PMC9472404; doi:10.1186/s12864-022-08878-x)
Supplement: Supplementary file 2 — Additional file 2: Table S1. Five biological replicates of four group samples used in this study. [file 12864_2022_8878_MOESM2_ESM.docx]

**Table S1. Five biological replicates of four group samples used in this study**

| **Uninfected sample** | **Infected sample** | **RNA-seq analysis** |
| --- | --- | --- |
| Single worm | Single worm +  *P. aeruginosa* | Conducted in this study |
| Bulk worms | Bulk worms +  *P. aeruginosa* | Conducted by us in a previous study (Sellegounder et al. 2019 Science Advances 5(11): eaaw4717) |
