# Supplementary material for: Using single-worm RNA sequencing to study C. elegans responses to pathogen infection
Source: BMC Genomics. 2022 Sep 14;23:653. doi: 10.1186/s12864-022-08878-x (PMC9472404; doi:10.1186/s12864-022-08878-x)
Supplement: Supplementary file 3 — Additional file 3: Table S2. Tissue-specific genes detected in single-worm samples. [file 12864_2022_8878_MOESM3_ESM.docx]

**Table S2. Tissue-specific genes detected in single-worm samples**

|  | **Hypodermis** | **Intestine** | **Muscle** | **Neurons** |
| --- | --- | --- | --- | --- |
| Reference (kaletsky et al. 2018 PLoS Genetics 14(8): e1007559) | 584 | 519 | 426 | 867 |
| Single-worm replicate 1 | 580 (99.3%) | 443 (85.4%) | 398 (93.4%) | 807 (93.1%) |
| Single-worm replicate 2 | 582 (99.7%) | 471 (90.8%) | 410 (96.2%) | 852 (98.3%) |
| Single-worm replicate 3 | 580 (99.3%) | 465 (89.6%) | 407 (95.5%) | 821 (94.7%) |
| Single-worm replicate 4 | 582 (99.7%) | 462 (89.0%) | 408 (95.8%) | 855 (98.6%) |
| Single-worm replicate 5 | 582 (99.7%) | 481 (92.7%) | 417 (97.9%) | 859 (99.1%) |
